# Population diversity, admixture, and demographic trend of the Sumba Ongole cattle based on genomic data

**DOI:** 10.5713/ab.23.0289

**Published:** 2023-11-02

**Authors:** Pita Sudrajad, Hartati Hartati, Bayu Dewantoro Putro Soewandi, Saiful Anwar, Angga Ardhati Rani Hapsari, Tri Satya Mastuti Widi, Sigit Bintara, Dyah Maharani

**Affiliations:** 1 Faculty of Animal Science, Universitas Gadjah Mada, Yogyakarta, 55281, Indonesia; 2 Research Center for Animal Husbandry, Research Organization for Agriculture and Food, National Research and Innovation Agency (BRIN), Bogor, 16911, Indonesia; 3 Research Center for Applied Zoology, Research Organization for Life Sciences and Environment, National Research and Innovation Agency (BRIN), Bogor, 16911, Indonesia; 4 Indonesian Research Institute for Animal Production, Indonesian Agency for Agricultural Research and Development, Ministry of Agriculture, Bogor, 16002, Indonesia

**Keywords:** Admixture, Demography, Diversity, Genom, Sumba Ongole Cattle

## Abstract

**Objective:**

Sumba Ongole (SO) cattle are valuable breed due to their important role in the development of Indonesian cattle. Despite rapid advances in molecular technology, no genomic studies on SO cattle have been conducted to date. The aim of this study is to provide genomic profile related to the population diversity, admixture, and demographic trends of SO cattle.

**Methods:**

Genomic information was gathered from 79 SO cattle using the Illumina Bovine SNP50 v3 Beadchip, and for comparative purposes, additional genotypes from 209 cattle populations worldwide were included. The expected and observed heterozygosity, inbreeding coefficient, pairwise fixation indices between-population, and Nei's genetic distance were examined. Multidimensional scaling, admixture, and treemix analyses were used to investigate the population structure. Based on linkage disequilibrium and effective population size calculations, the demographic trend was observed.

**Results:**

The findings indicated that the genetic diversity of SO cattle was similar to that of other indicine breeds. SO cattle were genetically related to indicines but not to taurines or Bali cattle. The study further confirmed the close relationship between SO, Ongole, and Nellore cattle. Additionally, a small portion of the Ongole mixture were identified dominant in the SO population at the moment. The study also discovered that SO and Bali cattle (*Bos javanicus*) could have been ancestors in the development of Ongole Grade cattle, which corresponds to the documented history of Ongolization. Our finding indicate that SO cattle have maintained stability and possess unique traits separate from their ancestors.

**Conclusion:**

In conclusion, the genetic diversity of the SO cattle has been conserved as a result of the growing significance of the present demographic trend. Consistent endeavors are necessary to uphold the fitness of the breed.

## INTRODUCTION

Sumba Ongole (SO) cattle (*Bos indicus*) have historically played a significant role in Indonesian cattle development. As the name implies, this cattle breed was developed from Ongole cattle bred on Sumba Island - Indonesia [1]. The high cost of importation and the increasing scarcity of superior Ongole cattle in India have prompted the Ongole cattle breeding program since 1914 in Sumba Island [2]. Subsequently, SO cattle breed has spread throughout Indonesia, especially on the island of Java. This process was dubbed "Ongolization", where these cattle have been used for grading up program [3]. In 1936, a strict regulation was issued stating that SO cattle were being spread through the "Sumba Contract" system. Furthermore, male local cattle had to be castrated, while females were mated with SO bulls [4]. However, many superior SO bulls were later sold outside of the region, causing genetic erosion and quality degradation [1]. Since then, other cattle breeds have been imported to Sumba Island and crossed with SO cattle in order to improve their performance [5]. According to statistics, the beef cattle population in Sumba was expected to be around 43 thousand head by 2022 [6].

A thorough understanding of livestock diversity and population structure is required for maintaining genetic variation and is crucial for developing future breeding strategies, and the use of molecular technology for this purpose is gaining popularity [7]. Previously, the genetic diversity of SO cattle has been investigated through various analyses utilizing low-density microsatellite markers and mitochondrial DNA markers [8,9]. The revolution in molecular approach and the increasing availability of a large number genome-wide single nucleotide polymorphisms (SNPs) of animals have transformed them into the most important markers, and they are now intended to be used for genetic studies [10,11]. Genomic markers have been used to study diversity in Indonesian local cattle in the worldwide population context [12], however SO cattle had not yet been included. By conducting genomic studies on SO cattle, will supplement previous knowledges and hypotheses, as these cattle play a significant role in the formation of Ongole Grade, a valuable cattle breed with the widest distribution in Indonesia [3,13].

This study therefore aimed to investigate the genomic profile of SO cattle. Our current study represents the first comprehensive genome-wide analysis for this population by optimizing the genotypes obtained using the Bovine50SNP beadchip (Illumina Inc., San Diego, CA, USA). To better understand SO population ancestry referring to its historical context, we added genome datasets of pure Ongole cattle and other white-coated cattle from India previously published by Dixit et al [14]. The findings of our study will provide novel insights into the population diversity, admixture, and demographic trend of Indonesian SO cattle, which could be used as fundamental consideration in future breeding strategies to maintain breed fitness.

## MATERIALS AND METHODS

### Ethical statement

All animal procedures were reviewed and approved by the Research Ethics Committee at Faculty of Veterinary Medicine, Universitas Gadjah Mada No.0120/EC-FKH/Eks./2022. Genotypes for the worldwide cattle breeds were obtained from previously published data [14,15], and therefore, no ethical approval was required.

### Genomic data sources and quality controls

This study utilized genomic data of SO cattle in Sumba island, consisting of 79 animals with medium density SNPs. Animals were collected from three SO cattle populations on East Sumba island, which had similar conditions and climate. The genomic data was obtained from DNA which were extracted from the blood samples. DNA extraction was conducted at the animal genetics laboratory of the Indonesian Research Institute for Animal Production in Bogor, following the procedure outlined in the Wizard® Promega isolation kit method. DNA evaluations, i.e. concentration quantification and purity, were performed using Picogreen and NanoDrop devices, respectively. DNA samples were deemed to be of sufficient quality if the concentration was at least 20 ng/μL and the absorbance ratio at 260 and 280 nm was more than 1.8. Appropriate samples were then genotyped using the Illumina BovineSNP50 v3 Beadchip. These DNA evaluations and genotyping process were finished by the service of Macrogen Inc. in Republic of Korea. We added genotypes of 209 animals consisted of 11 cattle populations which were selected from previously published genome datasets by Dixit et al [14] and Decker et al [15] to support the analyses in our study, i.e. Bali, Ongole Grade, Ongole, Nellore, Hariana, Tharparkar, Brahman, Limousine, Simmental, Hereford, and Shorthorn. The last two populations, in particular, were only used in the admixture analysis to determine whether a true Brahman cross (BX) mixture exists in the SO cattle population. The detail of datasets information were summarized in Table 1.

Quality control procedures were performed using PLINK v1.9 software [16] to ensure the reliability and accuracy of the data. SNP variants would be maintained if the Hardy–Weinberg Equilibrium (HWE) value was not <1×10^−4^. Any samples with a call rate of less than 90% were removed, as low call rates can indicate poor DNA quality or technical issues during genotyping. We also excluded any SNPs with a call rate of less than 90% or a minor allele frequency of less than 0.01, as these could indicate genotyping errors or non-polymorphic markers. Only autosomal SNPs with known genomic position based on UMD3.1 assembly and found in all populations were used in the subsequent analyses.

### Genetic diversity, population structure, and admixture analysis

This study began by calculating several diversity parameters, including expected heterozygosity (*H_e_*), observed heterozygosity (*H_o_*), inbreeding coefficient (*F_IS_*), pairwise fixation indices between-population (*F_ST_*), and Nei’s genetic distance to assess the genetic diversity of the SO and the comparators cattle populations. PLINK v1.9 [16] and R v4.1.0 [17] software were used to calculate these parameters. A genetic relationship matrix (GRM) of SO cattle was estimated using GCTA v1.93.2 [18]. A diagonal GRM value referring to the inbreeding of the animal itself and off-diagonal value referring to relationship between animals in the population.

This study also investigated the population structure using two different clustering methods: multi-dimensional scaling (MDS) and admixture analyses, which were performed using PLINK v1.9 [16] and fastSTRUCTURE [19], respectively. To determine the optimal number of subpopulations in the studied populations, the admixture analysis was ran using various estimated ancestral populations (referred to as K values). The cross-validation (CV) analysis was performed to select a minimum error value for K. Both clustering results were plotted in R v4.1.0 [17] to get understandable visualization. TreeMix v1.12 [20] was used to conduct extensive analysis of the relationships between cattle populations. A phylogenetic tree model was generated by positioning Bali cattle as root. The *threepop* command line was used to perform *f3* statistical analyses introduced by Reich et al [21] to identify possible traces of introgression. As possible introgression events of populations B and C into population A, three-population statistical models (A, B, and C) with a significant negative value for both the *f3* statistic and the Z-score were chosen.

### Estimation of linkage disequilibrium and effective population size

The cattle population's demographic history was reflected in the estimated current and former effective population sizes (*N_e_*). Sved and Hill [22] explained how past *N_e_* was estimated from the linkage disequilibrium (LD) value bases on the following equation: E(*r*^2^) = 1/(1+4*N_e_c*). LD was annotated as *r*^2^ before *N_e_* to measure the correlation of alleles at two loci and *c* was a recombination fraction in Morgan's unit. To estimate LD and *N_e_*, the default PLINK v1.9 [16] and SNeP v1.0 [23] approaches were used, respectively. The --*r2* command was used to get the LD value of SNP pairs, and the --*ld- window*-*r2* command was used to get reports for all pairs. The *N_e_* analysis was performed by using the standard input parameters in GONE approach [24] to infer a population's recent demographic history from SNP data of a small sample of individuals. Following the estimated times in the horizontal ordinate, the historical *N_e_* values were plotted using R v4.1.0 [17].

## RESULTS

### Population structure and diversity

The genotyping process using the Illumina BovineSNP50 v3 Beadchip yielded a total of 53,218 SNPs on SO cattle. The observed genotyping call rate was 99.28%. Due to the quality control process, 28% of the variants were removed, leaving only 38,310 SNPs to be merged with the other cleaned genotypes of other cattle breeds (Table 1). Combined file consisted of 22,652 variants which have been encountered on 288 animals from 12 cattle breeds. Variability of the samples in SO population was proven by the negative off-diagonal variances in the GRM analysis, i.e. −0.0131. SO cattle showed commensurate level of both *H_e_* and *H_o_* with the other indicines, where the position is lower than taurines and higher than *Bos javanicus* (Bali cattle). Ongole Grade cattle had the highest *H_e_* and *H_o_* among indicines. Discovered in all populations, the *H_o_* was slightly higher than the *H_e_*. Likewise with the *F_IS_* of SO cattle, the value (−0.21) was almost the same as other indicines. Moreover, the *F_IS_* of cattle populations observed in this study were all negative.

An initial investigation into genetic diversity is summarized in the pairwise *F_ST_* and Nei's genetic distance matrix (Table 2). This matrix clarified the distinctions and differences between SO and other cattle breeds. SO cattle were close to indicines but far from taurines and Bali cattle. Among indicines, Nellore cattle were the closest to Ongole from India (*F_ST_* 0.0025; Nei 0.0061), while SO cattle were the farthest (*F_ST_* 0.0074; Nei 0.0096). However, SO cattle had the shortest distance to Ongole cattle than to the others.

### Population cluster and ancestries

**Figure 1:**
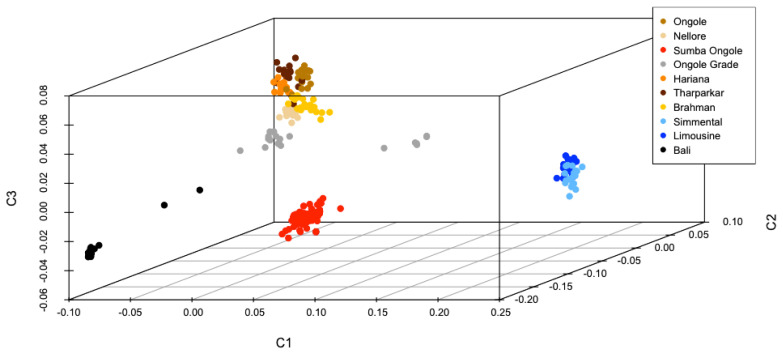
Multi-dimensional scaling plot provide an overview of the position of Sumba Ongole cattle againts other indicines (Ongole, Nellore, Ongole Grade, Hariana, Tharparkar, Brahman), taurines (Simmental, Limousine), and *Bos javanicus* (Bali) clusters.

**Figure 2:**
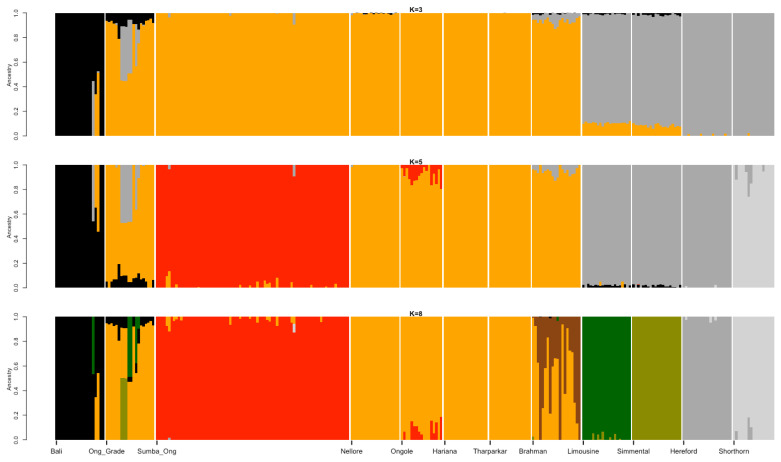
Admixture diagram depicts the estimated ancestry models composed on every individual in the populations. Mixture with K = 8 was chosen as the sensible model due to the minimum cross-validation error (0.523; Supplementary S1). The dominant mixture in Sumba Ongole cattle resembled a small portion of the Ongole cattle ancestry pattern.

Best illustration of the cattle genetic cluster could be explained by the MDS plot (Figure 1). A three-dimensional plot was used to clarify SO cattle position among indicines, taurines, and Bali cattle (*Bos javanicus*) coordinate. Interestingly, SO cattle populations tend to assemble in one colony but still near to indicines cluster, while Ongole, Nellore, Ongole Grade, Hariana, Tharparkar, and Brahman were close to one another. The admixture diagram (Figure 2) also shows the results of a more detailed cluster analysis. Based on the minimum CV error calculation (Supplementary Figure S1), K = 8 was chosen as the sensible model in this analysis. SO cattle could clearly be distinguished from other indicines, but the majority of their mixtures were similar to those found in Ongole cattle from India. Furthermore, the admixture with K = 3 was still displayed because it can be used to describe the sub species clusters, namely *Bos javanicus* (black color), *Bos indicus* (orange color), and *Bos taurus* (gray color).

**Figure 3:**
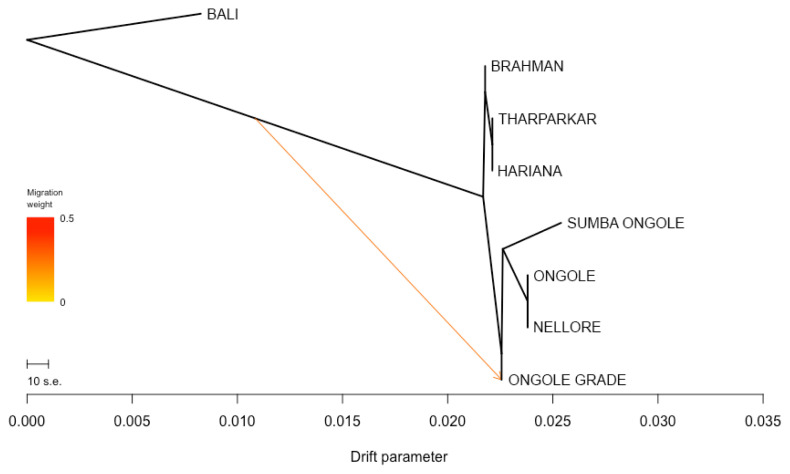
Phylogenetic tree of Sumba Ongole cattle among indicines with Bali cattle (*Bos javanicus*) as root. Sumba Ongole cattle were definitely associated with Ongole and Nellore cattle. Only one migration edge was observed, originating from Bali, and heading to Ongole Grade cattle.

The phylogenetic tree was developed specially for indicines and Bali cattle positioned as a root (Figure 3). Nellore and Ongole cattle from India were directly linked in the tree and SO cattle were closely related to those branch, while Ongole Grade cattle were in line after SO branch. The other indicines, i.e. Brahman, Tharparkar, and Hariana were in the different direction. Moreover, only one migration edge was observed, originating from the direction of Bali to Ongole Grade cattle. The *f3* statistic was generated and the most significant results marked by negative value of both *f3* statistics and Z-score were summarized in Table 3. This analysis only detect the possible introgression of Bali cattle (*Bos javanicus*) and indicines into Ongole Grade cattle. Here, SO cattle were the most likely candidate representing indicine ancestry (*f3* statistic −0.0055; Z-score −11.4887). We looked more closely at the *f3* statistics in which the Population A was SO cattle. This step was completed in order to identify the cattle breeds that have an impact to the SO population. Despite the fact that the values of both *f3* statistic and Z-score were not negative, we select the combination of three-population statistical models with the lowest value. As a result, we identified that Ongole or Nellore (indicines originated from India) and Brahman cattle were the populations that suitable in the columns of Population B and C, respectively.

### Demographic trends

**Figure 4:**
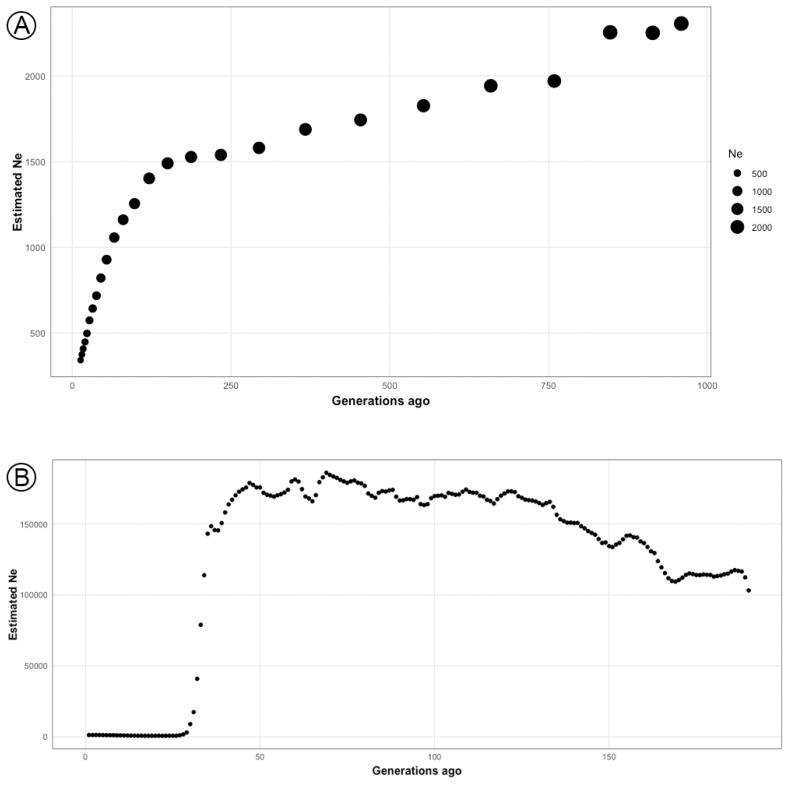
The historical effective population size (*N_e_*) estimation resulted from SNeP (A) and GONE (B) softwares. Both A and B reflect demographic changes over past generations of Sumba Ongole cattle. The similar patterns of dramatic *N_e_* decline were identified, i.e., at 54 to 13 (A) and 34 to 26 (B) generations ago (Supplementary Table S1).

An estimation of LD values based on *r*^2^ equation were done for all of SNP pairs and depicted following interallelic distances (Supplementary Figure S2). The observed LD values were high over a short allele distance and decayed steadily as the allele distance increased, while the average LD of adjacent markers (~100 kb) was 0.16±0.25. The historical *N_e_* was calculated based on the LD value across the genome and was used to represent demographic changes in SO cattle population (Figure 4; Supplementary Table S1). Results from the SNeP software started the *N_e_* estimation from 13th generation ago, while in GONE started from more recent time, i.e. 1st generation ago. Given in the similar generation time, both analyses were resulted in different values of *N_e_*, e.g. *N_e_* values in 13th generation ago were 922.48 and 342 for GONE and SNeP, respectively. However, both GONE and SNeP were detect similar pattern of dramatic Ne decline at almost in the simultaneously generation time estimates, i.e. GONE at 34 to 26 generations ago and SNeP at 54 to 13 generations ago. Moreover, according to the GONE estimation results, the values of *N_e_* from the 25th generation ago to the current generation were stable with a slight increase.

## DISCUSSION

Historical events in the development of cattle in Indonesia have resulted in the formation of unique local cattle populations, one of which is the SO cattle. Despite historical records indicating that the SO cattle have an ancestral connection to the Ongole breed from India, our investigations have revealed genetic characteristics in the SO population that differentiate it from its parent population. Thus, the off-diagonal GRM value was negative which means that each individual in the population was not genetically related [18]. Moreover, the amount of variant that remained after the quality control process is very common and feasible in other cattle breeds (Table 1). However, we used only 22,652 SNPs found in all cattle breed populations studied, ensuring that all analysis parameters can be applied equally to each population.

In terms of heterozygosity, SO cattle have a comparable value with other indicines (Table 1). It has been consistently reported that the heterozygosity value of the *Bos indicus* sub species is in the midst of *Bos taurus* and *Bos javanicus* [12]. However, some argued that *Bos indicus* should have a high level of heterozygozity. This is most likely explained by the ascertainment bias, which causes the heterozygozity level of indicine to be underestimated [25]. In contrast to the previous statement, it should be noted that there are a limited number of appropriate bulls available in the area where indicine cattle frequently reared [26], which may account for the genetic erosion and low heterozygozity. Only favorite bulls are used in mating, and this has an impact on heterozygozity which decreases over time. Moreover, the *H_o_* was reported higher than *H_e_* indicating that there are polymorphisms in the population. It also suggests that the population has an excess of heterozygotes. The ratio of heterozygous alleles to homozygous alleles can be a factor of deviation in the HWE, causing the appearance of a negative value in *F_IS_* [27], as found in this study. Low *F_IS_* value suggested a beneficial effect of regulated genetic inbreeding in the examined populations.

The measurements of genetic divergence between SO and the comparator populations in this study were proven in our consecutive analyses. The *F_ST_* and Nei's genetic distance analyses (Table 2) indicate that SO cattle are genetically closer to indicines than taurines and *Bos javanicus*, but among indicines, this cattle breed were not most similar (in term of allele frequencies) to Ongole cattle native to India. We noticed that Nellore cattle have approximately closest genetic relationship to Ongole cattle. In this study, Ongole Grade cattle were the population shown to have a close distance to both Ongole and Nellore. While in our previous hypothesis, Ongole Grade population in Kebumen regency were proposed as the maintained pure Ongole cattle breed in Indonesia [12]. Three-dimensional MDS plot clearly depicts the description of SO cattle position between the coordinates of indicines, taurines, and *Bos javanicus*. Even though the SO cattle were classified as zebu, they do not have a coordinate point that is close to the other indicines (Figure 1), whereas SO cattle were clearly on the same branch as Ongole and Nellore cattle in the phylogenetic tree (Figure 3).

The results of the ancestry estimation can explain such SO conditions. Only a small portion of the Ongole cattle ancestry pattern is now the dominant mixture in SO cattle (Figure 2). This is possible because the Ongole breeding, and purification program were carried out on the island of Sumba over a long period of time [28]. The dominant characters that SO cattle have today may be the result of adaptation process and selection patterns that have occurred within their environment. In the admixture analysis, we were unable to identify the BX mixture in the SO population. Therefore, the study was failed to verify the information provided by Sumadi and Siliwolu [5] that a cross between SO and BX cattle had occurred on Sumba island. This could be because BX cattle were only introduced outside of the SO breeding area. Further study must be carried out directly including BX cattle genome data, keeping in mind that we currently only use Brahman, Hereford, and Shorthorn cattle genotypes as a mixture representation of BX cattle.

Furthermore, we were able to provide genetical evidence that SO cattle were used to form Ongole Grade cattle. The results of the *f3* statistical analysis significantly estimated that SO cattle were an introgressive population to Ongole Grade cattle (Table 3). This finding supports historical confirmation that SO cattle were used in Indonesia's Ongolization program [3]. In addition, the study has also detected evidence confirming the occurrence of introgression from Ongole cattle to SO cattle, but its value has since declined. These findings align with the results obtained from the admixture analysis. As a result, the study verifies that, despite the SO cattle are descendants of India's Ongole breed, they have exhibited stability and possess unique characteristics that differentiate them from their ancestors. The demographic trend analysis provides interesting points. Patterns of drastic *N_e_* decline in SO population detected by both SNeP and GONE approaches (Figure 4) were common and found in most cattle populations. Breed formation, importation of animals, the isolation of population, merging, and eventually breed separation can all be attributed to the extraordinary changes in the demographic pattern [29]. If we look attentively, we can observe that the initial decline of *N_e_* occurred during the formation of SO. While the stable curve condition with slight increase obtained from GONE analysis since the 25th generation ago up to the present may reflect SO cattle development efforts on the island of Sumba, to maintain genetic variation.

## CONCLUSION

The genetic analyses show that the SO cattle population in Indonesia has distinct characteristics that set it apart from its Indian ancestor, the Ongole cattle. This study provides genetic evidence for historical records showing that Ongole Grade cattle originated through introgression from the SO population, and the SO cattle have undergone adaptation and selection processes over time. Despite the decline in effective population size, efforts have been made to maintain its genetic variation.

## Figures and Tables

**Table 1:** Summary of the genomic datasets information

Cattle breed	Sub species	Samples	Initial SNPs	Cleaned SNPs	He	Ho	FIS
Sumba Ongole	*B. indicus*	79	53,218	38,310	0.31	0.39	−0.21
Ongole Grade^1)^	*B. indicus*	20	42,885	30,210	0.33	0.43	−0.23
Ongole^2)^	*B. indicus*	17	470,703	20,016	0.32	0.41	−0.20
Nellore^1)^	*B. indicus*	20	42,885	30,210	0.31	0.40	−0.22
Hariana^2)^	*B. indicus*	18	470,703	22,454	0.31	0.40	−0.21
Tharparkar^2)^	*B. indicus*	17	470,703	22,454	0.31	0.40	−0.22
Brahman^1)^	*B. indicus*	20	42,885	30,210	0.33	0.42	−0.22
Bali^1)^	*B. javanicus*	20	42,885	30,210	0.14	0.21	−0.21
Limousine^1)^	*B. taurus*	20	42,885	30,210	0.41	0.56	−0.32
Simmental^1)^	*B. taurus*	20	42,885	30,210	0.41	0.56	−0.33
Hereford^1)^	*B. taurus*	20	42,885	39,275	0.42	0.59	−0.35
Shorthorn^1)^	*B. taurus*	17	42,885	39,275	0.39	0.56	−0.34

SNP, single nucleotide polymorphisms; *H_e_*, expected heterozygosity; *H_o_*, observed heterozygosity; *F_IS_*, inbreeding coefficients.

1) Decker et al [15].

2) Dixit et al [14].

**Table 2:** Pairwise *F_ST_* (lower diagonal) and Nei’s genetic distances between populations (upper diagonal)

Cattle breed	Sumba Ongole	Ongole Grade	Ongole	Nellore	Hariana	Tharparkar	Brahman	Bali	Limousine	Simmental
Sumba Ongole	0	0.0102	0.0096	0.0107	0.0102	0.0109	0.0106	0.0492	0.0313	0.0331
Ongole Grade	0.0083	0	0.0076	0.0073	0.0077	0.0087	0.0076	0.0394	0.0273	0.0291
Ongole	0.0074	0.0040	0	0.0061	0.0083	0.0092	0.0088	0.0497	0.0312	0.0331
Nellore	0.0089	0.0041	0.0025	0	0.0084	0.0093	0.0085	0.0446	0.0304	0.0322
Hariana	0.0082	0.0043	0.0047	0.0051	0	0.0072	0.0074	0.0446	0.0317	0.0331
Tharparkar	0.0088	0.0052	0.0055	0.0059	0.0038	0	0.0083	0.0462	0.0322	0.0340
Brahman	0.0088	0.0042	0.0050	0.0052	0.0039	0.0046	0	0.0439	0.0284	0.0301
Bali	0.0495	0.0399	0.0512	0.0452	0.0453	0.0469	0.0443	0	0.0587	0.0601
Limousine	0.0303	0.0242	0.0278	0.0275	0.0284	0.0287	0.0253	0.0597	0	0.0145
Simmental	0.0323	0.0262	0.0299	0.0295	0.0301	0.0308	0.0272	0.0613	0.0099	0

**Table 3:** The most significant *f3* statistics and the top results related to Sumba Ongole

Population A	Population B	Population C	*f3* statistics	Standard error	Z-score
A. Significant *f3* statistics					
Ongole Grade	Bali	Sumba Ongole	−0.0055	0.0005	−11.4887
Ongole Grade	Bali	Ongole	−0.0044	0.0008	−5.6271
Ongole Grade	Bali	Tharparkar	−0.0030	0.0008	−3.7589
Ongole Grade	Nellore	Bali	−0.0028	0.0007	−3.7935
Ongole Grade	Bali	Brahman	−0.0019	0.0007	−2.5775
Ongole Grade	Bali	Hariana	−0.0008	0.0008	−1.0848
B. Top observed results related to Sumba Ongole cattle					
Sumba Ongole	Ongole	Brahman	0.0104	0.0011	9.7436
Sumba Ongole	Nellore	Brahman	0.0105	0.0011	9.6011

## Data Availability

The datasets analysed during the current study are available from the corresponding author on reasonable request.

## SUPPLEMENTARY MATERIAL

**Supplementary Figure S1**. Plot of the cross-validation error estimation on the admixture analysis.

**Supplementary Figure S2**. Linkage disequilibrium decay of Sumba Ongole cattle.

**Supplementary Table S1**. Linkage disequilibrium (*r*^2^) and the historical effective population size (*N**_e_*) of Sumba Ongole cattle resulted in SNeP and GONE softwares
