# Supplementary material for: Population diversity, admixture, and demographic trend of the Sumba Ongole cattle based on genomic data
Source: Anim Biosci. 2023 Nov 2;37(4):591–9. doi: 10.5713/ab.23.0289 (PMC10915215; doi:10.5713/ab.23.0289)
Supplement: Supplementary file 1 [file ab-23-0289-Supplementary-Fig-S1.pdf]

Supplementary Data

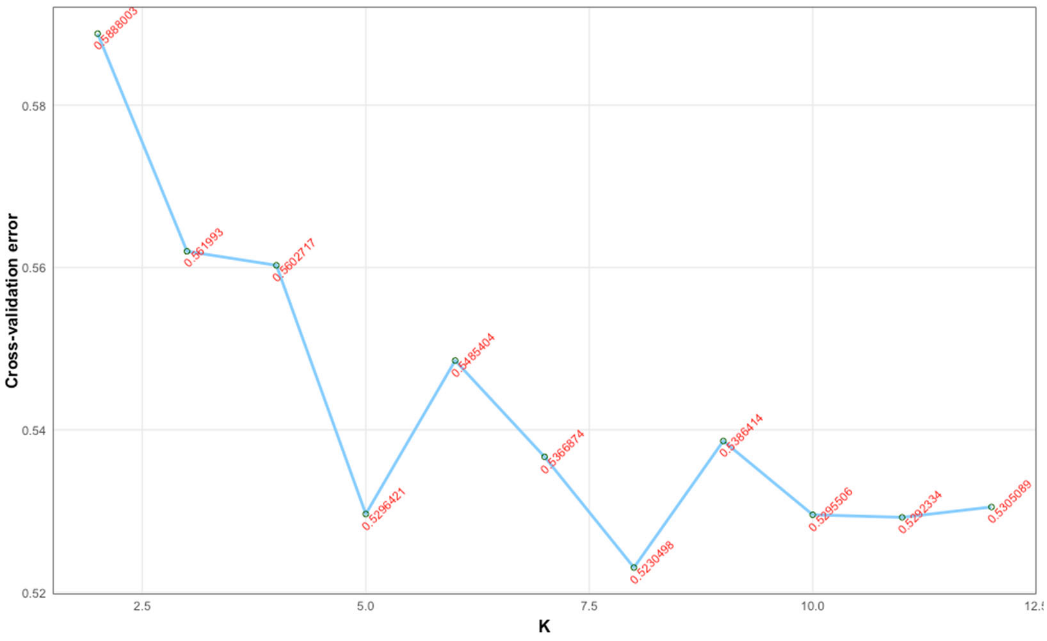

Supplementary Figure S1. Plot of the cross-validation error estimation on the admixture analysis. The minimum error value (0.523) was found for K=8.

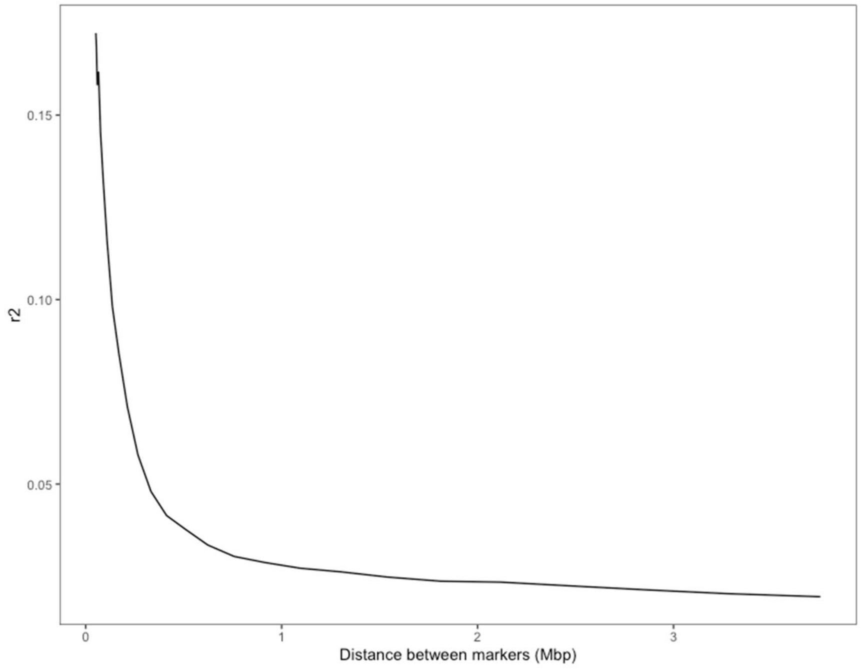

Supplementary Figure S2. Linkage disequilibrium decay of Sumba Ongole cattle. Over a short allele distance, the observed linkage disequilibrium (r^2) values were high and steadily decreased as the allele distance increased.
