# Supplementary material for: Population diversity, admixture, and demographic trend of the Sumba Ongole cattle based on genomic data
Source: Anim Biosci. 2023 Nov 2;37(4):591–9. doi: 10.5713/ab.23.0289 (PMC10915215; doi:10.5713/ab.23.0289)
Supplement: Supplementary file 3 [file ab-23-0289-Supplementary-Table-S1.pdf]

Supplementary Table S1. Linkage disequilibrium (r^2) and the historical effective population size (N_e) of Sumba Ongole cattle resulted in SNeP and GONE softwares

GONE software		SNeP software		
Generation Ago	N_e	Generation Ago	r^2 ± SD	N_e
1	1,322.17	13	0.02 ± 0.03	342
2	1,322.17	15	0.02 ± 0.03	375
3	1,322.17	17	0.02 ± 0.04	408
4	1,322.17	20	0.02 ± 0.04	448
5	1,242.27	23	0.02 ± 0.04	498
6	1,219.15	27	0.02 ± 0.04	574
7	1,196.96	32	0.02 ± 0.04	643
8	1,183.13	38	0.03 ± 0.04	718
9	1,099.78	45	0.03 ± 0.05	821
10	1,062.92	54	0.03 ± 0.05	928
11	1,027.21	66	0.03 ± 0.05	1,057
12	1,004.18	80	0.03 ± 0.06	1,161
13	922.48	98	0.04 ± 0.07	1,255
14	879.94	121	0.04 ± 0.07	1,402
15	823.35	150	0.05 ± 0.09	1,490
16	799.07	187	0.06 ± 0.10	1,527
17	775.94	234	0.07 ± 0.12	1,539
18	775.84	294	0.09 ± 0.14	1,580
19	769.63	367	0.10 ± 0.16	1,688
20	766.80	454	0.12 ± 0.18	1,743
21	787.76	553	0.13 ± 0.19	1,826
22	771.60	659	0.15 ± 0.21	1,942
23	785.88	759	0.16 ± 0.22	1,970
24	823.95	847	0.16 ± 0.22	2,254
25	822.21	914	0.17 ± 0.23	2,251
26	855.20	959	0.17 ± 0.23	2,305
27	1,137.85			
28	1,796.77			

GONE software			SNeP software		
Generation Ago	N_e		Generation Ago	$r^2 \pm$ SD	N_e
29	3,134.27				
30	8,989.03				
31	17,472.1				
32	40,914.3				
33	78,853.1				
34	113,826				
35	143,056				
36	148,347				
37	145,635				
38	145,410				
39	150,551				
40	158,056				
